# Hyaluronic acid-based nano drug delivery systems for breast cancer treatment: Recent advances

**DOI:** 10.3389/fbioe.2022.990145

**Published:** 2022-08-24

**Authors:** Yufeng Jia, Siwen Chen, Chenyu Wang, Tao Sun, Liqun Yang

**Affiliations:** ^1^ Department of Breast Medicine, Liaoning Cancer Hospital, Cancer Hospital of China Medical University, Shenyang, China; ^2^ Center for Molecular Science and Engineering, College of Science, Northeastern University, Shenyang, China; ^3^ NHC Key Laboratory of Reproductive Health and Medical Genetics (China Medical University), Liaoning Research Institute of Family Planning (The Reproductive Hospital of China Medical University), Shenyang, China; ^4^ Department of Information Management, Cancer Hospital of China Medical University, Liaoning Cancer Hospital, Shenyang, China

**Keywords:** hyaluronic acid, breast cancer, anticancer drugs, nanoparticles, drug delivery system (DDS)

## Abstract

Breast cancer (BC) is the most common malignancy among females worldwide, and high resistance to drugs and metastasis rates are the leading causes of death in BC patients. Releasing anti-cancer drugs precisely to the tumor site can improve the efficacy and reduce the side effects on the body. Natural polymers are attracting extensive interest as drug carriers in treating breast cancer. Hyaluronic acid (HA) is a natural polysaccharide with excellent biocompatibility, biodegradability, and non-immunogenicity and is a significant component of the extracellular matrix. The CD44 receptor of HA is overexpressed in breast cancer cells and can be targeted to breast tumors. Therefore, many researchers have developed nano drug delivery systems (NDDS) based on the CD44 receptor tumor-targeting properties of HA. This review examines the application of HA in NDDSs for breast cancer in recent years. Based on the structural composition of NDDSs, they are divided into HA NDDSs, Modified HA NDDSs, and HA hybrid NDDSs.

## 1 Introduction

Cancer is one of the significant causes of death in the world. Cancer can occur in many body areas, such as the liver, lungs, prostate, or breasts. Breast cancer is the most common cancer among women, accounting for nearly 30% of all cancers (Liyanage et al., 2019). According to statistics, there were 22,61,419 new breast cancer cases worldwide last year, along with the highest mortality rate of all types of cancer in women (684,996 deaths in 2020), seriously threatening the health status of women (Dubey et al., 2022). Although breast cancer is the most common type of cancer in women, it can be treated if detected and diagnosed in its early stages (Waks and Winer, 2019). However, if the cancer cells metastasize and invade other tissues through the blood and lymphatic system, it becomes significantly more challenging to treat, increasing the mortality rate rapidly (Lou et al., 2016).

Chemotherapy is an effective way to treat breast cancer. This strategy involves treating breast cancer by using chemotherapeutic drugs to prevent the mitosis and growth of cancer cells (Sharma et al., 2010). So far, many anti-cancer drugs such as paclitaxel, Adriamycin, tamoxifen, docetaxel, and methotrexate have been approved for breast cancer treatment (Nomura, 1996; Perez, 1998; Colleoni et al., 2002; Smith et al., 2002; Jordan, 2014). Unfortunately, breast cancer is highly resistant to chemotherapy drugs, the poor solubility of chemotherapeutic drugs in the body, the short duration of circulation of drug molecules in the bloodstream, the limited choice of oncologic agents, and the potential severe drug side effects have greatly limited the role of chemotherapeutic drugs in the treatment of breast cancer (Luo et al., 2019). Furthermore, prolonged treatment can also cause multidrug resistance (MDR), leading to treatment failure and tumor relapse. Therefore, in recent years, nano drug delivery systems (NDDSs) based on nanotechnology have been extensively studied to overcome the shortcomings of chemotherapeutic drugs in breast cancer treatment.

Compared to conventional medical systems, NDDS has distinct advantages, including the ability to increase the solubility of anti-breast cancer drugs, control and prolong drug release, achieve targeted drug delivery by enhancing the permeability to the tumor and the enhanced permeability and retention effect (EPR) and reduce the side effects of drugs at other sites (Hu and Zhang, 2009). So far, a limited number of NDDSs have been approved by the Food and Drug Administration (FDA) for clinical use, such as Doxil, albumin-bound paclitaxel nanoparticles (Abraxane), poly (lactic acid) (PLA), micelles-based paclitaxel (Genxol), and paclitaxel-loaded liposomes (Weissig et al., 2014). Unfortunately, NDDS regimens with active targeting capabilities are under evaluation and have not yet been approved. The most significant obstacle encountered in developing NDDS is that the material must have good biocompatibility. Therefore, a series of naturally degradable polymers with excellent biocompatibility have received widespread interest (Lokeshwar et al., 2014; Wu et al., 2018; Narmani and Jafari, 2021).

Hyaluronic acid (HA) is a natural linear mucopolysaccharide composed of alternating repeats of D-glucuronic acid and nacetyl-D-glucosamine, the main component of the extracellular matrix (Fraser et al., 1997). The excellent hydrophilicity of HA reduces adsorption (corona) and permeation with proteins, thereby promoting the long-term circulation and stability of DDS composed of HA *in vivo* (Mizrahy et al., 2014). In addition, HA is an anionic polymer (pKa = 3–4) (Cadete and Alonso, 2016), enabling it to interact with cationic polymers, surfactants, and lipids to form a variety of nanostructures. In terms of composition, HA has carboxyl and hydroxyl groups and an N-acetyl group, offering unlimited potential for further modification (Burdick and Prestwich, 2011).

It is well known that the CD44 receptor is one of the many transmembrane glycoproteins that have great potential in achieving active targeting therapy. Meanwhile, CD44 is a primary receptor for HA (UNDERHILL, 1992). Standard CD44 (CD44s) is widely present in normal cells; HA binds to receptors and contributes to angiogenesis, wound healing, tissue hydration, and cell signaling in the extracellular matrix (Necas et al., 2008; Oh et al., 2010; Mattheolabakis et al., 2015; Wickens et al., 2017). CD44 is expressed at low levels on the surface in healthy cells, such as epithelial cells, hematopoietic cells, and neuronal cells (Huang and Chen, 2019). In contrast, variant CD44 (CD44v) is overexpressed on the surface of various cancer cells, including breast, squamous, ovarian, and colon carcinomas (Kim et al., 2018). Notably, CD44v has a higher affinity for HA than CD44s (Misra et al., 2011). In addition, HA has excellent hydrophilicity, good biocompatibility, biodegradability, and non-immunogenicity (Lapcık et al., 1998). The surface charge of HA-NDDSs is typically negative, which facilitates blocking the clearance of nano-carriers by the reticuloendothelial system (RES) (Xiao et al., 2011). In addition, nano-carriers are selectively transferred into cancer cells through the EPR effect and active targeting of CD44 receptors (Oh et al., 2010). Furthermore, HA has good biocompatibility, biodegradability, and non-immunogenicity. Therefore, HA-NDDSs have been extensively studied in the field of drug delivery.

**FIGURE 1:**
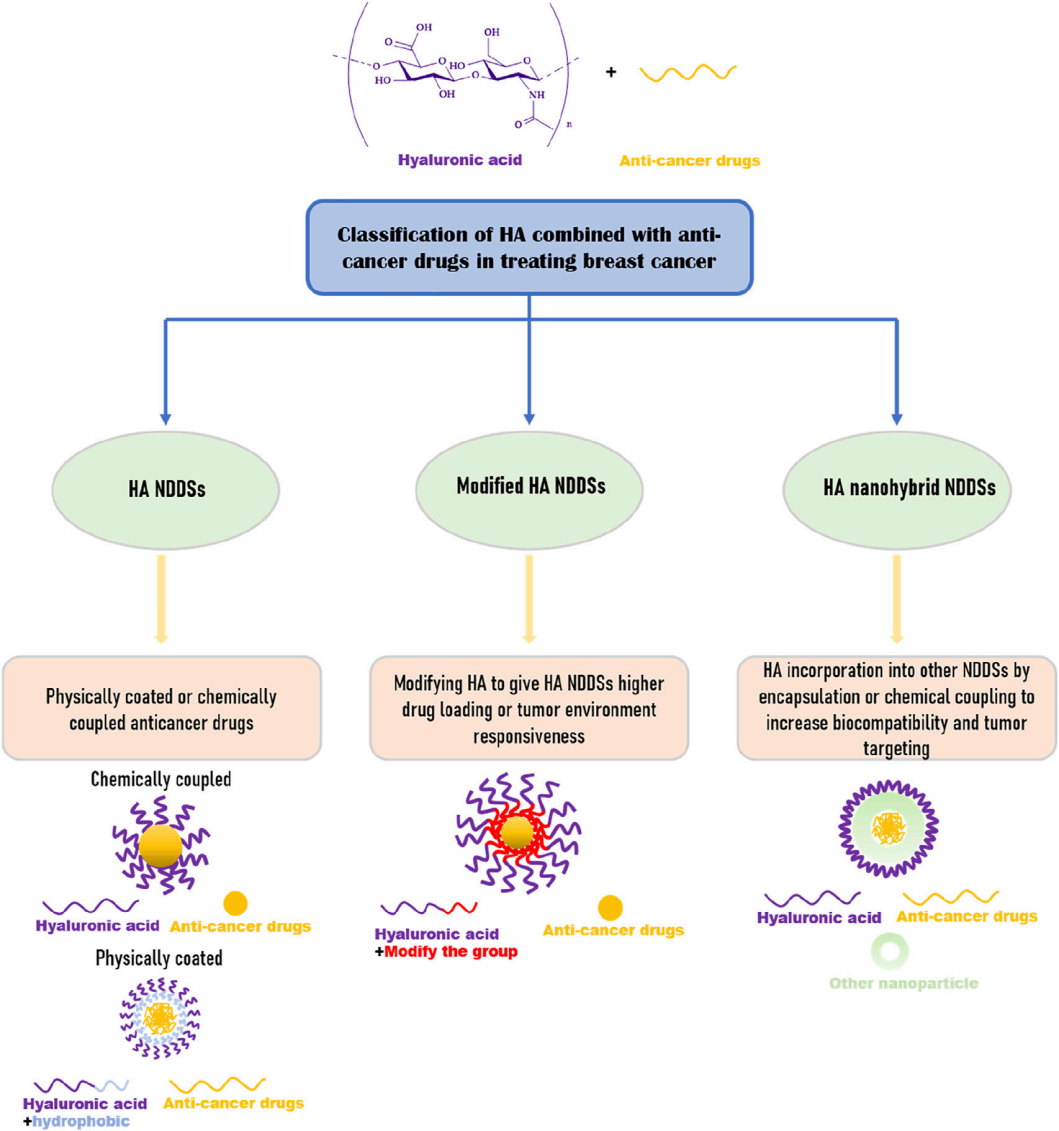
Classification of hyaluronic acid NDDSs in breast cancer.

We focus on the review of HA as NDDSs in breast cancer therapy in recent years, including HA NDDSs, Modified HA NDDSs, and HA nanohybrid NDDSs (Figure 1).

## 2 HA NDDSs

Chemotherapeutic agents used to treat breast cancer generally have disadvantages such as low water solubility, slow delivery, and non-specific biodistribution and targeting, hence restricting their clinical applications (Dheer et al., 2017).

HA with anti-cancer drugs provides better hydrophilicity and stability and improves drug absorption efficiency by targeting CD44 receptors while reducing the side effects of drugs on healthy cells (Yang et al., 2013; Tran et al., 2017). Table 1 summarizes NDDSs with HA-coated or modified nano-drug in recent years, including NDDSs drug type, size, drug loading capacity, etc.

**TABLE 1:** Characteristics of HA NDDSs.

Component	Formulation	Therapeutics	Size (nm)	%DLC	Indication	Status	References
Lapatinib-HA-NCs	Nanoparticle	Lapatinib	288.8 ± 17.4 PDI:0.272 ± 0.022	**—**	MDA-MB-231 4T1	*In vivo*	Agrawal et al. (2018)
HA@ Curcumin -NC	Nanoparticle	Curcumin	161.85 ± 1.70 PDI: 0.25 ± 0.02	—	MDA-MB-231 4T1	*In vivo*	Ji et al. (2020)
HA- Cationic naproxen P-NPs	Nanoparticle	Naproxen	297.0 ± 6 PDI:0.113 ± 0.017	HA adsorption:46%	MCF-7 HepG2	*In vitro*	Espinosa-Cano et al. (2021)
HA- Shikonin -Lip	Liposome	Shikonin	173 ± 5 PDI: 0.14 ± 0.02	3.3 ± 0.1	MDA-MB-231	*In vivo*	Meng et al. (2022)
HA-Lip-17-hydroxy-jolkinolide B	Liposome	17-hydroxy-jolkinolide B	130.8 ± 1.9	3.6 ± 0.1	4T1	*In vivo*	Liu et al. (2021)
HA nanohydrogel of quercetin	Nanogels	Quercetin Everolimus	211.3 ± 5.3 PDI:0.1 ± 0.014	∼12.09	MCF-7	*In vitro*	Quagliariello et al. (2017)
HA-ionic-triphenylphosphonium - Doxorubicin	Nanoparticle	Doxorubicin	∼257 PDI:0.096	TTP-DOX:31.4	MCF-7/ADR	*In vivo*	Liu et al. (2018a)

Lapatinib (LPT) is an anti-cancer drug approved to treat advanced HER2 + breast cancer (Castellino et al., 2012). Agrawal et al. prepared LPT-HA-NCs by HA encapsulating nanocrystalline LPT to enhance the therapeutic efficacy and reduce drug side effects in triple-negative breast cancer (TNBC). It was shown that the anti-cancer activity of LPT-HA-NCs was superior to that of LPT-NCs and free LPT through the active targeting ability of CD44 receptor, which is characteristic of HA, and had lower toxic side effects. LPH-HA-NCs showed 83.32% tumor reduction after 28 days of anti-tumor treatment and effectively inhibited lung metastasis. LPH-HA-NCs at low doses exhibit significant therapeutic effects, thereby suggesting a strategy for minimizing the dose for the treatment of TNBC (Agrawal et al., 2018).

Curcumin (Cur) is one of the dietary polyphenolic compounds with anti-cancer effects (Hong et al., 2017). To improve the surface hydrophilicity of curcumin nanocrystals (Cur-NC) and increase the drug release time, Ji et al. modified the surface of Cur-NC using HA to prepare HA@Cur-NCs. According to the data, HA@Cur-NCs significantly improved the bioavailability of Cur compared to free drugs and Cur-NC, prolonged the retention time of Cur *in vivo*, and showed better anti-cancer effects in mice loaded with 4t1 with negligible systemic side effects. HA@Cur-NCs provide a new alternative for the future clinical treatment of breast cancer (Ji et al., 2020).

**FIGURE 2:**
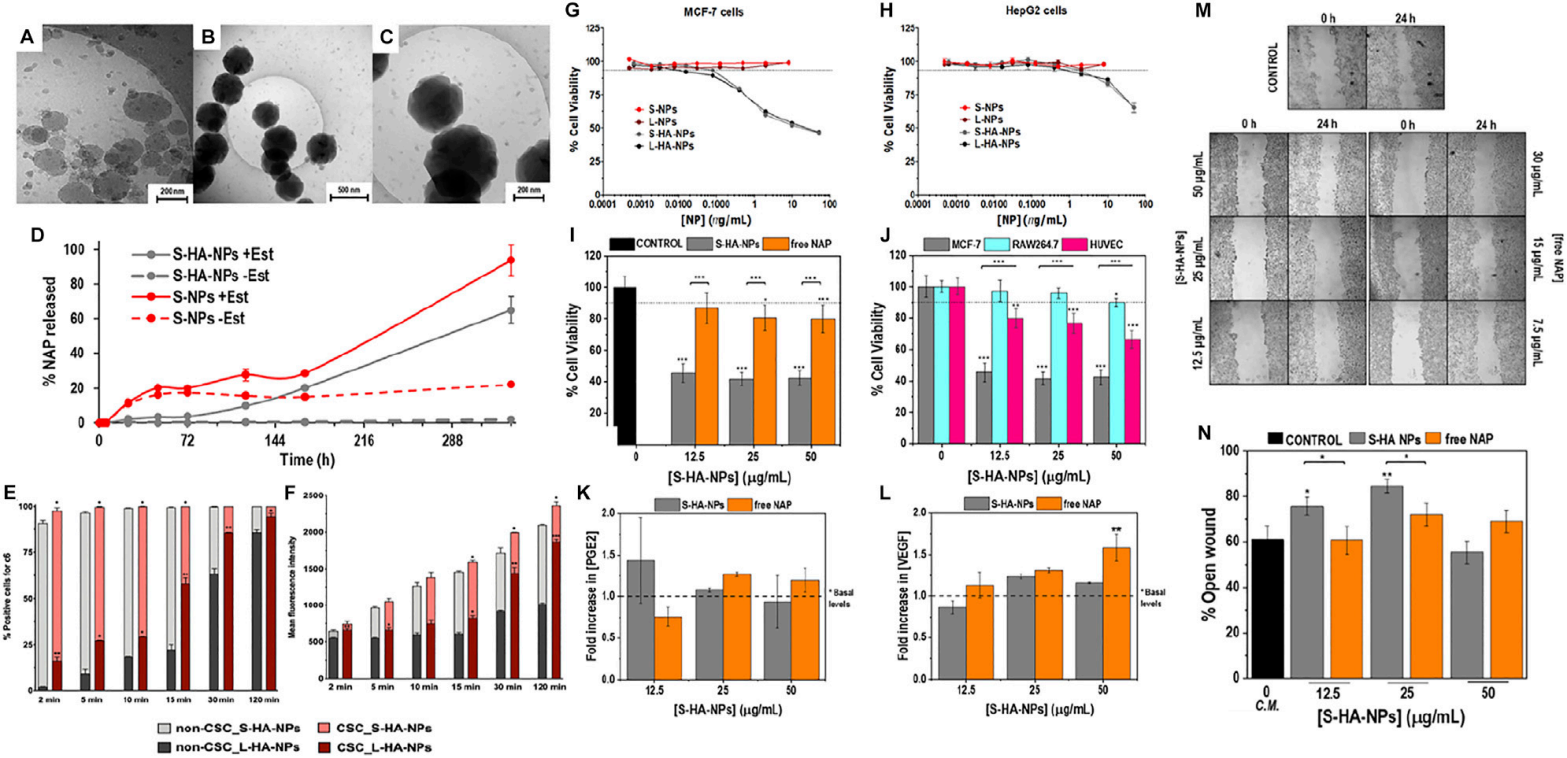
**(A)** Uncoated NPs, without HA covering **(B)** HA-NPs at low magnification, **(C)** HA-NPs at higher magnification. **(D)** Naproxen release kinetics from HA-coated or uncoated NPs. **(E)** Percentage of positive cells for c6 and **(F)** the mean fluorescence intensity per cell (t-student, *p < 0.05, **p < 0.01, ***p < 0.001). *In vitro* cytotoxicity of HA-NPs in cells with differential expression of CD44. **(G)** Percentage of cell viability of MCF-7 cells (high expression of CD44) after 72 h of treatment with different concentrations. **(H)** Cell viability experiments in HepG2 cells (low expression of CD44) using the same NPs. Cell viability assays by Alamar Blue in MCF-7, RAW264.7 and HUVEC cells treated with S-HA-NPs. **(I)** Percentage of viable MCF-7 cells relative to control (culture media of MCF-7 cells) after 72 h of treatment with different concentrations. **(J)** Percentage of cell viability in respect to controls (cells treated with culture media) after 72 h of treatment with different concentrations of S-HA-NPs(Statistical analysis was performed by one-way ANOVA test with *p < 0.01, **p < 0.05 and ***p < 0.001). ELISA quantification of **(K)** PGE2 and (L)* VEGF released by MCF-7 cells after 72 h of treatment (Statistical analysis was performed by one-way ANOVA test with **p < 0.05). Wound healing assay in S-HA-NPs or free NAP treated MCF-7 cells. Effect of different concentrations of S-HA-NPs or free NAP on MCF-7 migration *in vitro*: **(M)** Inverted microscope images (20-fold magnification) of the wound at the beginning of the assay (0 h) and 24 h post-scratching and **(N)** Percentage of open wound after 24 h of treatment when compared to the original wound size (Statistical analysis was performed by one-way ANOVA with *p < 0.01 and **p < 0.05). Reproduced with permission from ref (Liu et al., 2018a). CC BY 4.0. Copyright 2021 The Authors.

According to previous studies, chronic inflammation is closely related to carcinogenesis (Arias et al., 2007). Anti-inflammatory drugs are emerging as new candidates in the treatment and prevention of cancer. Naproxen (NAP) is a well-known non-steroidal anti-inflammatory drug, and its derivatives have been well demonstrated for treating breast cancer (Deb et al., 2014). Espinosa-Cano et al. synthesized HA-NPs from cationic NAP-bearing polymeric NPs (NPs) with excellent anti-inflammatory ability by electrostatically imparting HA coatings. Based on data analysis, the drug release from NPs with HA coating was more stable and linear in the study of NAP release kinetics. Furthermore, regarding the CD44-targeted cellular uptake ability, smaller NPs were shown to be internalized more rapidly in MCF-7 cancer cells than medium to larger NPs. For both types of NPs, regardless of size, faster and more intense internalization of HA-NPs in CD44^+^ CSC, hence CD44-HA interactions can be considered an excellent vehicle to improve the efficiency of anti-cancer drugs against CSC. Cytotoxicity and scratch assays demonstrated that HA-NPs not only had a significant targeting effect on RAW264.7, HUVEC, and MCF-7 in terms of toxicity but also had a better inhibitory effect on MCF-7 than free NAP. The system also allowed a reduced dose of NAP for pro-apoptotic and anti-migration activity. Overall, HA-NPs can potentially improve the treatment of advanced breast cancer (Espinosa-Cano et al., 2021) (Figure 2).

Reactive oxygen species (ROS) is a significant indicator for regulating biological functions (Perillo et al., 2020). ROS level in cancer cells is higher than that in healthy cells, which ROS can realize to promote tumor proliferation and metastasis. One of the ways chemotherapeutic drugs induce apoptosis in cancer cells is to enhance oxidative stress in cells by increasing intracellular ROS levels (Yang et al., 2018a). Therefore ROS inducers that effectively cause apoptosis of cancer cells through oxidative stress can be potential candidates for cancer chemotherapy. Shikonin (SHK) is a bioactive natural naphthoquinone isolated from the roots of Lithospermum erythrorhizon, which has demonstrated good bioactivity in cancer therapy and anti-inflammation and wound healing (Guo et al., 2019). Meng et al. utilized hyaluronic acid encapsulated SNK liposomes to obtain HA-SHK-Lip to treat triple-negative breast cancer (TNBC). As a result, the internalization ability and retention time of HA-SHK-Lip in MDA-MB-231 cells was significantly higher than free SHK and SHK-Lip. HA-SHK-Lip effectively increased intracellular oxidative stress by effectively upregulating ROS levels in MDA-MB-231 cells and decreasing intracellular GSH (53.6% in the control group) through ROS caused apoptosis and thus exhibited excellent anti-tumor ability. This study provides an effective therapeutic strategy to combat TNBC by enhancing cellular oxidative stress (Meng et al., 2022).

Recently, an active anticancer substance, 17-hydroxy-jolkinolide B (HJB), a diterpenoid from the roots of *Euphorbia* Fischer Diana Steud, has attracted the interest of researchers. In clinical cancer therapy, low water solubility and bioavailability are the main factors limiting its application (Jian et al., 2018a; Jian et al., 2018b). Liu et al. first studied the effect of HJB on breast cancer inhibition. The HJB liposomes were encapsulated with HA by electrostatic adsorption to obtain HA-Lip-HJB, aiming to improve its hydrophilicity and bioavailability and enhance tumor targeting *in vivo*. The results showed that HA-Lip-HJB significantly improved the delivery of liposomes in 4T1 cells in response to the CD44 receptor. In addition, HA-Lip-HJB could inhibit the 4T1 cell migration of cancer cells by regulating EMT progression. Meanwhile, in tumor suppression tests, *in vitro* 3D tumor spheroids decreased in size to 5.9% of the original size after 9 days, and *in vivo* tumor suppression was finally 75.6% after 15 days, which was higher than that of free HJB and Lip-HJB, evidencing the excellent anti-tumor ability of HA-Lip-HJB. It could also inhibit the migration and colonization of tumor cells in the lung. Therefore, the HA-Lip-HJB prepared by the research group provides a potential strategy for treating metastatic breast cancer (Liu et al., 2021).

Quercetin is a natural candidate for novel anti-inflammatory and anti-cancer agents; recently, it has attracted attention for its anti-tumor activity (İkizler et al., 2007). Everolimus is an oral rapamycin analog used to treat breast cancer (Royce and Osman, 2015). In 2017, Quagliariello et al. prepared a HA nanogel based on CD44-targeted loaded quercetin for co-administration with everolimus derivatives against hormone-responsive human breast cancer cells MCF7. The objective was to combine natural molecules with chemotherapy to reduce drug doses, thereby reducing side effects and improving treatment outcomes (Nam et al., 2016). In the research, the nano-carriers had good biosafety, and the CD44-mediated endocytosis of the nanohydrogels in human breast cancer cells MCF-7 showed significant internalization of up to 24 h incubation and exhibited higher cytotoxicity than free quercetin against human breast cancer cells MCF-7. In addition, the nano-carriers had good biosafety and could significantly internalize within 24 h of incubation due to CD44-mediated endocytosis of the nanohydrogels in human breast cancer cells MCF-7. Furthermore, the quercetin-loaded HA nanogels exhibited higher cytotoxicity than free quercetin against human breast cancer cells MCF-7. According to the data, the combination of everolimus and quercetin hyaluronic acid hydrogel produced a highly synergistic effect on MCF-7 cells, showing a good inhibition of MCF-7 cells by low concentration administration (10 nM everolimus, 2 mg/ml quercetin HA) with fewer toxic side effects (approximately 60% reduction in the IC50 of everolimus at 72 h). This combination therapy has a profound future in treating breast cancer (Quagliariello et al., 2017).

Adriamycin (DOX) has also been studied to be modified and coupled with HA to form NDDSs for drug delivery targeting breast cancer. Liu et al. used triphenylphosphine (TPP) to bind with HA through ionic bonding, to form a supramolecular self-assembled structure HA-ionic-TPP-DOX between TPP-DOX and HA. HA-ionic-TPP-DOX possessed the ability to target mitochondria inside breast cancer cells through the CD44 receptor targeting of HA and the property of TPP to accumulate inside mitochondria. HA-ionic-TPP-DOX in MCF-7 cells can accumulate more DOX in mitochondria, increasing reactive oxygen species (ROS) levels and decreasing mitochondrial membrane potential, resulting in superior anti-tumor effects in MCF-7/ADR cells in zebrafish. In conclusion, HA-ionic-TPP-DOX is promising in treating breast cancer (Liu et al., 2018a).

## 3 Modified HA NDDSs

By modifying the hydrophilic HA surface with hydrophobic tips (polymers or small molecules) and obtaining amphiphilic NDDSs by self-assembly (Ondreas et al., 2021). These NDDSs have a core-shell structure, with a hydrophilic shell to increase the stability of NDDSs in water for prolonged circulation and a hydrophobic core for drug encapsulation and sustained release. HA has excellent hydrophilic and CD44 receptor targeting properties without triggering the “accelerated blood clearance (ABC) phenomenon (Zhang et al., 2016). Table 2 presents the Modified HA NDDSs prepared by other polymers or small molecules modified HA in recent years.

**TABLE 2:** Characteristics of modified HA NDDSs.

Component	Formulation	Therapeutics	Size (nm)	%DLC	Indication	Status	References
HA-poly caprolactone	Polymersome	Doxorubicin	146.2 ± 9.6	3.6 ± 0.4	4 T1 MCF-7	*In vivo*	Shahriari et al. (2019)
DOX-HA-polylactic acid	Nanoparticle	Doxorubicin	123.9 ± 3.3 PDI:0.191 ± 0.030	DLE: 71.7 ± 3.8	4T1	*In vivo*	Deng et al. (2018)
Dox/HA-ss- ibuprofen	Micelles	Doxorubicin	∼120 PDI:0.2	—	4T1	*In vivo*	Chai et al. (2020)
DTX/HA-cys-docosahexaenoic acid/chlorin e6	Nanoparticle	Docetaxel	181.4 ± 1.8 PDI:0.241 ± 0.039	—	MCF-7	*In vivo*	Wang et al. (2021a)
HA-DTX-Dendron	Dendronized polymer	Docetaxel	122 ± 4 PDI:0.174	—	MDA-MB-231	*In vivo*	Wang et al. (2021b)
SFN/M-HA-SS-n-Tetradecanethiol	Nanoparticle	Sulforaphane	85.90 ± 3.49 PDI:0.13 ± 0.01	DLE:33.64 ± 1.33	MDA-MB-231	*In vivo*	Gu et al. (2021)
HA-DOX-cisplatin	Micelles	Doxorubicin, Cisplatin	∼80	—	4 T1	*In vivo*	Yu et al. (2020)
α1-acid glycoprotein-HA NPs	Nanoparticle	Doxorubicin	352 ± 22	—	MCF-7, MDA-MB-231	*In vitro*	Omar et al. (2022)

Recently, Shahriari et al. developed a novel hyaluronic acid-polycaprolactone (HA-PCL) nanopolymer, encapsulated doxorubicin (DOX) in HA-PCL, and studied the therapeutic index and biodistribution of HA-PCL-DOX for a 4T1 metastatic mouse breast cancer model. The obtained results confirmed that HA-PCL-DOX has a higher uptake rate than PEG-PCL-DOX and free DOX in 2 cell types (4T1 and MCF-7), owing to the targeting of the CD44 receptor. In addition, HA-PCL-DOX was shown to be non-significantly toxic to mice organs and to have optimal tumor suppression, effectively prolonging the survival rate of mice. Accordingly, HA-PCL may be considered a potential ideal candidate for DOX delivery in breast cancer treatment (Shahriari et al., 2019).

Efficient delivery of drugs to tumor cells and eventual complete elimination of the tumor is the ultimate purpose of cancer treatment. Unfortunately, due to high interstitial fluid pressure and the multilayered character of tumor cells, anti-cancer drugs are challenging to deliver inside tumors (Jain, 1990; Heldin et al., 2004). The tumor penetrating peptide-iRGD facilitates the penetration of anti-cancer drugs and NDDSs into tumor tissue and can inhibit tumor metastasis (Sugahara et al., 2010; Sugahara et al., 2015). In order to enhance the effectiveness of NDDSs in breast cancer treatment, a DOX-loaded HA-PLA (DOX-HA-PLA) NDDS combined with an iRGD drug delivery system was developed by Deng et al. Their group studied the inhibitory effect of the combined system on the growth and metastasis of breast cancer. The results indicated that DOX-HA-PLA eliminated the ABC phenomenon compared to DOX-PLA and the ability to be taken up by 4T1 cells through specific HA-CD44 interactions. *In vivo* and tumor penetration studies showed significantly better tumor accumulation and lung distribution of DOX-HA-PLA compared to DOX-PLA controls. When co-administered with iGRD, DOX-HA-PLA accumulation at tumor and lung metastasis sites was dramatically increased. The combined administration remarkably enhanced the tumor suppressive effect and survival time of the mice, and no significant lung metastasis was observed. The combined delivery strategy of DOX-HA-PLA and iGRD enhances DOX inhibition of breast cancer tumor growth and metastasis, providing a promising new strategy for breast cancer treatment (Deng et al., 2018).

Precursor drug-polymer micelles (PMs) can improve the aqueous solubility and bioavailability of anticancer drugs (Biswas et al., 2016). However, the lack of tumor targeting and environmentally responsive drug release have limited their further clinical application (Yin et al., 2018). PMs can be used to realize combination therapy by co-delivery different drugs (Ha et al., 2018). Therefore, preparing PMs-NDDSs with targeting ability is a potential therapeutic strategy to treat breast cancer.

Glutathione (GSH) is an antioxidant widely found in mammals. GSH levels are higher in tumor cells than in extracellular and normal cells (Liu, 2019). Therefore, redox sensitivity has been extensively studied as a stimulating condition for drug release (Akita et al., 2013). In 2020, Chai et al. synthesized a hyaluronic acid-ibuprofen prodrug (HA-ss-BF) with a redox response by binding ibuprofen (BF) to HA *via* disulfide bonds. HA-ss-BF could be a drug delivery carrier for DOX by self-assembly (DOX/HA-ss-BF). *In vitro*, DOX/HA-ss-BF showed excellent inhibition of metastasis (48h, ∼9.42%) and improved cell uptake through CD44 receptor mediation. In addition, HA-ss-BF effectively down-regulated the biological levels of COX-2 in 4T1 cells by CD44 receptor- and redox-response-triggered BF release. *In vivo*, DOX/HA-ss-BF showed excellent biosafety and remained capable of rapid accumulation at tumor sites *via* HA-CD44 interactions. In addition, Dox/HA-ss-BF had a good suppressive effect on 4T1 tumor growth (15d, ∼58.9%) and metastasis by reducing intracellular COX-2 levels through BF release and the obstruction of tumor growth *in situ* by DOX. DOX/HA-ss-BF shows potential for effective treatment of metastatic breast cancer and offers a novel direction for targeted combination drug delivery for breast cancer treatment (Chai et al., 2020).

In another study, HA was surface modified with docosahexaenoic acid (DHA) and chlorin e6 (Ce6) *via* disulfide bonding to obtain amphiphilic HA derivatives HA-cys-DHA/Ce6 (CHD) for encapsulation of doxorubicin (DTX) for targeted treatment of breast cancer to achieve combined chemotherapy and photodynamic therapy (PDT), and *in vivo* fluorescence imaging by Ce6 to provide aid in tumor diagnosis and treatment. DTX/CHD nanoparticles increased the uptake of MCF-7 cells through the CD44 receptor, thus reducing side effects and realizing redox response in tumor cells to enable the release of DTX and Ce6. Meanwhile, DTX and Ce6 exerted favorable anti-tumor effects under the action of NIR light. The combination therapy of chemotherapy and PDT was realized (Wang et al., 2021a).

In addition to the preparation of NDDSs using linear HA polymers, NDDSs obtained by coupling glycodendrons with HA to obtain dendronized polymers can further improve the drug-carrier interactions and effectively enhance the drug delivery efficiency (Zhang et al., 2020a). Wang et al. used HA-DTX coupling as a design idea, linking DTX to HA by a tetrapeptide with proteinase B response (GFLG) and a disulfide bond linking the glycoside to HA to obtain the branched and functional nanostructures HA-DTX-Dendron (HADD). Studies showed that the presence of glycodendrons enhanced the stability of HADD and promoted the accumulation in breast cancer tumors. The stimulatory response of GFLG and disulfide bonds in breast cancer cells increased the rate of DTX release, enabling HADD to demonstrate excellent anti-breast cancer tumor efficacy (Wang et al., 2021b).

The synthesis of GSH-responsive amphiphilic conjugates mostly requires tedious steps. More importantly, these amphiphilic conjugates are not morphologically stable enough after being highly diluted by liquids (Owen et al., 2012). Biomineralization provides a novel strategy for designing simple and stable GSH-responsive amphiphilic conjugates. Calcium phosphate (CaP), a mineral component with good biocompatibility, can be used to mineralize amphiphilic coupled nano-carriers. CaP mineralized nano-carriers can remain stable in a typical human environment (pH 7.4), but tumor-specific drug release can be realized in the weakly acidic tumor cell environment (pH 5–6.5) (Han et al., 2013). Gu et al. based amphiphilic hyaluronic acid-SS-tetradecyl conjugates (HA-SS-TA), mineralized by CaP to obtain M-HA-SS-TA for the delivery of sulforaphane (SFN) with anticancer efficacy for the suppression of breast cancer stem cells (BCSC). M-HA-SS-TA had excellent targeting ability to tumors *via* CD44 receptors and could release SFN rapidly to tumor ecological niche based on pH and redox responses. Therefore, SFN/M-HA-SS-TA exhibited better suppression of BCSC *in vitro* and *in vivo* compared to free SFN. Overall, M-HA-SS-TA provides a new strategy for both the preparation of amphiphilic nanoparticles and the treatment of breast cancer (Gu et al., 2021).

**FIGURE 3:**
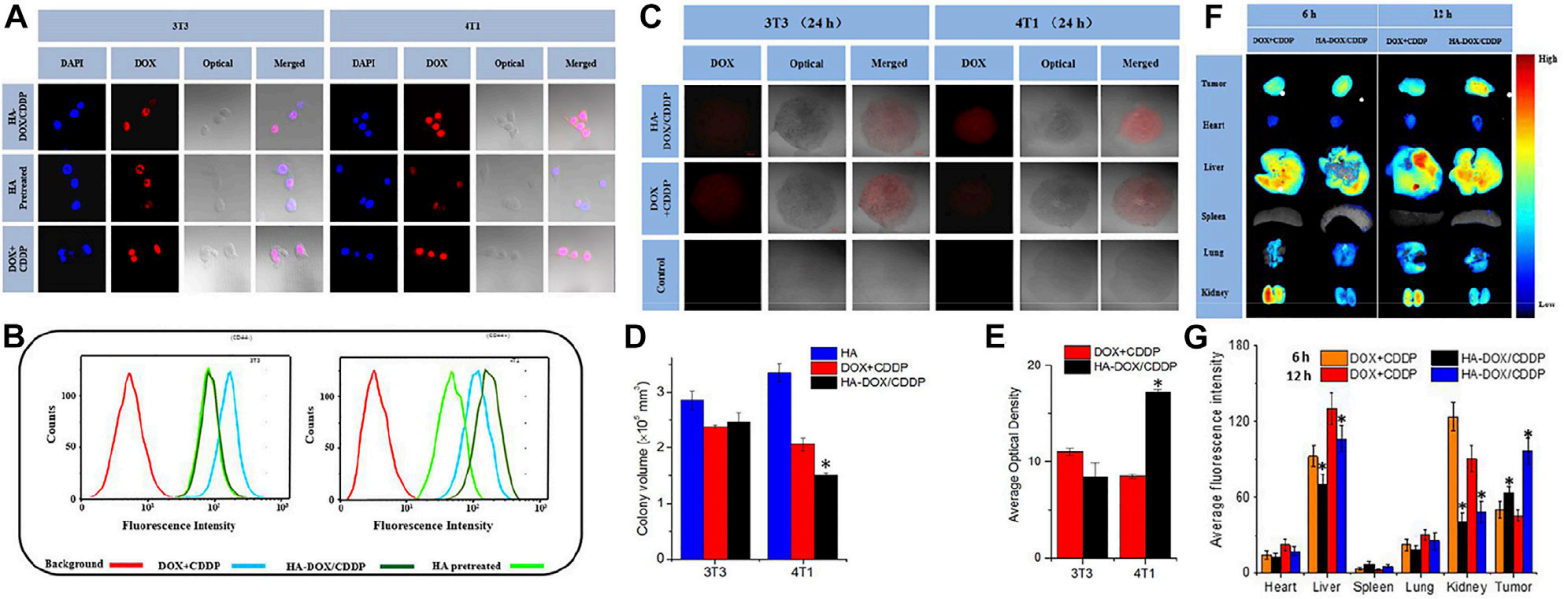
*In vitro* cellular uptake and intracellular DOX release in 3T3 and 4T1 cells. **(A)** Confocal laser scattering microscopy and **(B)** FCM analysis were performed on 3T3 and 4T1 cells following HA pretreatment, and treatment with free DOX + CDDP and HA-DOX-CDDP. *In vitro* multicellular spheroids in 3D suspension cultures. **(C)** Confocal laser scattering microscopy of 4T1 and 3T3 cell spheroids treated with HA, DOX + CDDP, and HA-DOX-CDDP for 24 h. **(D)** Colony volume and **(E)** fluorescence density analyses of 4T1 and 3T3 cell spheroids treated with HA, DOX + CDDP, and HA-DOX-CDDP for 24 h (*p < 0.05 compared with the DOX + CDDP group). *In vivo* DOX biodistribution. **(F)** *Ex vivo* fluorescence images of isolated organs and tumors at 6 or 12 h post-injection. **(G)** Semi-quantitative analysis of the mean fluorescence intensity in isolated organs and tumors at 6 or 12 h post-injection. Data are presented as the mean ± SD (*n* = 3) (*p < 0.05 compared with the DOX + CDDP group). Reproduced with permission from ref (Owen et al., 2012). CC BY 4.0. Copyright 2020 The Authors.

**FIGURE 4:**
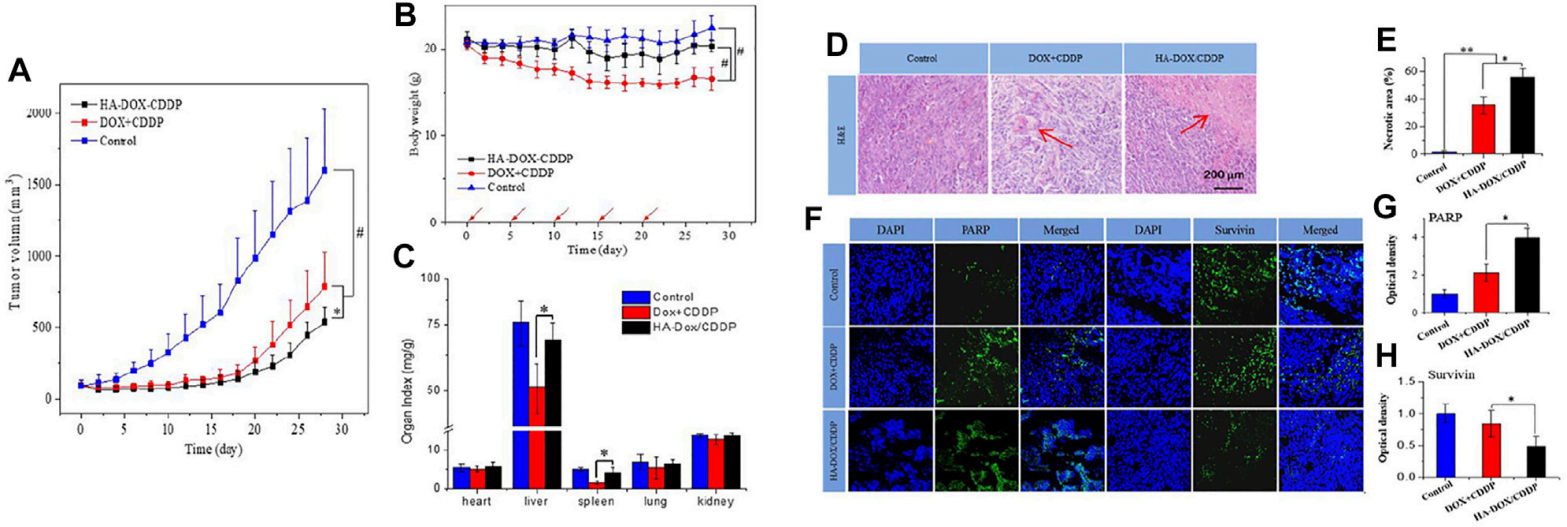
*In vivo* safety and antitumor efficacies. **(A)** Tumor volumes and **(B)** body weights of 4T1-xenografted mice after treatment with NS as the control, DOX + CDDP, or HA-DOX-CDDP. Red arrows showed the tail-veil injection time. **(C)** Organ coefficients of isolated organs in NS, DOX + CDDP, or HA-DOX-CDDP treated groups (*p < 0.05, #*p* < 0.001). Histopathology and immunofluorescence analyses. **(D)** Histopathological (H&E) analyses and **(E)** necrotic areas in H&E-stained tumor sections from 4T1-xenografted mice following treatment with NS as the control, DOX + CDDP, or HA-DOX-CDDP. Red arrows indicated the necrotic area. **(F)** Immunohistochemical (PARP and survivin) analyses of tumor tissue sections following treatment with NS as the control, DOX + CDDP, or HA-DOX-CDDP. **(G)** Relative optical densities of tumor sections showing PARP immunofluorescence. **(H)** Relative optical densities of tumor sections showing survivin immunofluorescence (Data are presented as the mean ± SD (*n* = 5). *p < 0.05, **p < 0.01). Reproduced with permission from ref (Owen et al., 2012). CC BY 4.0. Copyright 2020 The Authors.

In another facile strategy for preparing a pH-sensitive HA-based anti-breast cancer drug delivery system, HA-DOX-CDDP dual drug-loaded micelles were synthesized by cross-linking cisplatin (CDDP) with HA chelation and loading DOX by electron interaction. The pH sensitivity of HA-DOX-CDDP resulted in its significantly enhanced drug release efficiency under acidic conditions. In *in vitro* cell uptake studies, HA-DOX-CDDP showed a differentiated targeting effect on 3T3 and 4T1 cells and, in addition, showed higher internalization compared to the free drug (DOX + CDDP) and HA pretreatment groups. Therefore, HA-DOX-CDDP showed a better internalization effect as well as CD44 targeting. Moreover, HA-DOX-CDDP showed enhanced drug penetration in 3D 4T1 cell cultures. HA-DOX-CDDP has the best *in vivo* tumor suppression effect compared to other experimental groups while ensuring lower toxicity to systemic tissues and organs. Compared with the free drug group, mice treated with HA-DOX-CDDP tumor sections showed increased PARP expression and decreased survivin expression with sound anti-tumor effects (Yu et al., 2020) (Figures 3, 4).

The phenomenon of multidrug resistance (MDR) evasion is a capability possessed by most NDDSs but is powerless against the metastasis of inflammatory tumors (Vyas et al., 2014). The anti-inflammatory protein α1-acid glycoprotein (AGP) can suppress the expression of pro-inflammatory factors such as TNF-α and interleukins (IL-6) in the tumor microenvironment (Landskron et al., 2014). Therefore, Omar et al. prepared AGP-HA NPs by combining AGP and HA NPs for targeted delivery of DOX to suppress the growth and migration of tumor cells. The results showed that AGP-HA NPs could effectively inhibit the tumor migration of MCF-7 cells by suppressing inflammation. In addition, AGP-HA NPs could effectively deliver DOX to the nucleus of MDA-MB-231 cells and inhibit cell proliferation and induce apoptosis. In summary, AGP-HA NPs inhibit the ability of inflammation to attenuate breast cancer cell migration, providing a promising nanotherapeutic platform for the treatment of breast cancer (Omar et al., 2022).

## 4 HA hybrid NDDSs

In order to improve the effectiveness of breast cancer treatment, it is necessary to give some other advantages to NDDSs composed of HA, including high drug loading, more precise targeting, and more effective inhibition of cancer cell growth and metastasis, etc. The development of HA hybrid NDDSs composed of HA and other substances offers the possibility of better treatment of breast cancer. In recent years, HA hybrid NDDSs composed of HA in combination with other substances (both organic and inorganic) are shown in Table 3.

**TABLE 3:** Characteristics of HA hybrid NDDSs.

Component	Formulation	Therapeutics	Size (nm)	%DLC	Indication	Status	References
Mesoporous silica nanoparticle-HA-Curcumin	Nanoparticle	Curcumin	75–110	14.76	MDA-MB-231	*In vivo*	Ghosh et al. (2021)
Biodegradable mesoporous silica nanoparticle, HA	Nanoparticle	Doxorubicin, IR780	69.15 ± 3.4	DOX:1.1 ± 0.3 IR780: 9.4 ± 1.8	MCF-7	*In vivo*	Zhan et al. (2021)
Gold nanorods-HA- Folate-DOX	Nanoparticle	Doxorubicin	70.9 ± 1.4	7.1	MCF-7	*In vivo*	Xu et al. (2017)
AuNC@Bovine serum albumin-PTX-Indocyanine green@HA-NO_3 、_	Nanoparticle	Paclitaxel, Indocyanine green	195.6 ± 3.2 PDI:0.181 ± 0.012	PTX: 2.97% ± 0.05	4T1	*In vivo*	Liu et al. (2018b)
Cu(II)-Quercetin-Dextran-aldehyde/superparamagnetic iron oxide nanoparticle@HA	Nanoparticle	Quercetin, Cu(II)	—	0.3 ± 0.02 mg/ml	MDA-MB-231, HCC1395	*In vivo*	Cheng et al. (2021)
HA-graphene oxide-Metformin	Nanoparticle	Metformin	673.4 PDI: 0.235	—	MDA-MB-231	*In vitro*	Basu et al. (2021)
HA- carbon dots @p-4-Carboxybenzaladehyde-DOX	Nanoparticle	Doxorubicin	38.61 ± 14.30 PDI: 0.214	22.15%	4T1	*In vivo*	Li et al. (2020)
(DOX/levulan (5-aminolevulinic acid -AHCS/HA^HER2^)	Nanocomplexe	Doxorubicin levulan (5-aminolevulinic acid	∼140	ALA:29.38% DOX:11.47%	MCF-7	*In vitro*	Wang et al. (2019a)
NP^HER2^(DOX/Cisplatin)	Nanoparticle	Doxorubicin, Cisplatin	∼162	DOX:20.55 CDDP:9.53	MCF-7	*In vitro*	Wang et al. (2019b)
Dox,miR34a/Chitosan - linoleic acid/HA	Nanoparticle	Doxorubicin	180 ± 8.3 PDI:0.082 ± 0.03	13.5 ± 1.20	MCF-7/A	*In vivo*	Yang et al. (2021)
17α-Methyltestosterone/HA–chitosan–lipoic acid	Nanoparticle	17α-Methyltestosterone	280 ± 0.045 PDI:0.327 ± 0.002	10.30	BT-20, MCF-7	*In vitro*	Rezaei et al. (2020)
HA-Zein-Honokiol	Nanoparticle	Honokiol	∼200	DLE:88.1%∼93.6	4T1	*In vivo*	Zhang et al. (2020b)
HA-DOX/Poly (L-histidine)/R848	Nanoparticle	Doxorubicin, R848	∼200 PDI:0.215	DOX:∼6.5 R848:22.8	4T1	*In vivo*	Liu et al. (2018c)
Polypyrrole@Camptothecin-HA-IRDye800CW	Nanoparticle	Camptothecin Photothermal therapy (PTT)	86 ± 9.2	CPT-HA:2.2	4T1	*In vivo*	Sun et al. (2019)
HA/Polyethyleneimine	Nanoparticle	Docetaxel α-napthtoflavone	193.6 ± 3.1 PDI:0.169	DTX:1.14 ± 0.18 ANF:1.56 ± 0.20	MCF-7/1B1	*In vitro*	Zhang et al. (2019)
Hyaluronic Acid-Based Conjugate/D-α-Tocopheryl Poly (ethylene glycol) 1000 Succinate	Micelles	Doxorubicin, Quercetin	201.2 ± 2.36 329.83 ± 2.24	D-MM: 8.81 Q-MM: 2.46	MDA-MB-231/MDR1	*In vivo*	Liu et al. (2020)
HA-D-α-tocopherol succinate-(4-carboxybutyl)triphenyl phosphonium bromide/LPT	Nanoparticle	Lapatinib	207 ± 3 PDI:0.19 ± 0.03	DLE:84%	MDA-MB-231	*In vivo*	Lee and Cho, (2018)
E-selectin binding peptide-polyethene glycol-1-octadecylamine-HA-PTX/PTX	Micelles	Paclitaxel	102.6 ± 7.9 PDI:0.169	31.5	4TI	*In vivo*	Han et al. (2017)
TMX- Papain -HA-ss-lithocholic acid	Nanoparticle	Tamoxifen	367.5 PDI: 0.116	—	MCF-7	*In vivo*	Batool et al. (2020)
Cur@ZIF-8@HA	Nanoparticle	Curcumin	184.1 ± 13.2	9.6	4T1	*In vivo*	Yu et al. (2021)

### 4.1 HA hybrid with inorganic mineral

#### 4.1.1 Mesoporous silica

Mesoporous silica (MSN) has received widespread attention as an alternative material for synthesizing NDDS due to its large specific surface area, adjustable particle and pore size, excellent biocompatibility, and high drug encapsulation efficiency (Gao et al., 2020). Recently, Ghosh et al. bonded MSN and HA to synthesize HA-MSN nanohybrid NDDS, which combined CD44 receptor targeting ability and high drug delivery efficiency. By loading nanohybrid with curcumin (MSN-HA-C), the effect of MSN-HA-C in inhibiting triple-negative breast tumors was studied. The results showed that MSN-HA-C effectively inhibited the growth and migration of triple-negative breast cancer tumors in mice through induction of ROS, cell cycle arrest, and regulation of NF-κB and Bax-mediated apoptotic pathways. Compared with free curcumin, it has more efficient bioavailability and cellular uptake (Ghosh et al., 2021). In addition, Zhan et al. prepared bMID NPs with chemotherapeutic and photothermal therapeutic capabilities by equipping DOX and photothermal initiator IR780 based on HA and MSN. MID NPs could accumulate at the tumor site through CD44 receptors and effectively enhance the circulation time of the drug *in vivo* (Yue et al., 2013). bMID NPs showed an increased survival rate in mice compared to the free drug DOX and IR780 groups and favorable inhibition of breast cancer tumors by chemotherapy and photothermal therapy. Thus bMID NPs provide novel nanoplatforms for breast cancer treatment (Zhan et al., 2021).

#### 4.1.2 Gold nanoparticles

Gold nanorods (GNRs) are promising nanomaterials in biomedicine. Au NRs are frequently used for photothermal therapy (PTT) in the treatment of breast cancer due to the strong surface plasmon resonance absorption in the near-infrared spectral region (NIR) while converting NIR to heat with an efficiency close to 100% (Wang et al., 2011; Maestro et al., 2014). Furthermore, rod-shaped nanoparticles exhibit better *in vivo* circulation time and aggregation at the tumor site compared to spherical ones (Chauhan et al., 2011; Barua et al., 2013). However, the non-specific delivery of GNRs limits their application in PTT. To maximize the PTT effectiveness of GNRs in breast cancer cells, it is essential to improve the targeted delivery capacity and *in vivo* circulation duration of Au NRs. In 2017, Xu et al. developed a pH and NIR dual-responsive nanoplatform GNRs-HA-FA-DOX based on HA and GNRs for actively targeted chemotherapy and synergistic photothermal treatment of breast cancer. HA was modified by folic acid (FA), used to modify GNRs, and carried DOX through an unstable hydrazone bond with a pH response. The results showed that trifunctionalized HA increased the morphological stability of GNRs-HA-FA-DOX, increased tumor targeting, and prolonged *in vivo* circulation time, where the addition of FA further promoted the endocytosis of GNRs and DOX by MCF-7 cells compared to the CD44 targeting of HA. *In vitro* and *in vivo*, GNRs-HA-FA-DOX showed excellent chemotherapy/PTT synergism, outperforming chemotherapy or PTT alone. *In vivo* studies, mouse tumors were eliminated after 20 d of treatment, with no recurrence and severe side effects. In conclusion, GNRs-HA-FA-DOX shows excellent potential in breast cancer treatment, maximizes the effect of combined chemotherapy/photothermal treatment, and provides new ideas for designing multifunctional nanocarriers (Xu et al., 2017).

Cancer diagnosis is another issue besides treatment, and combining diagnosis with a treatment simultaneously is the future trend in treating cancer (Khandelia et al., 2015). While most NDDSs designed to treat breast cancer lack diagnostic functions, some imaging functions need to be given to monitor and evaluate the treatment effect. In recent years, gold nanoclusters have been increasing attention for applications such as fluorescence imaging and diagnostics (Zhang et al., 2015a; Zhou et al., 2016). In a previous study, Xie et al. synthesized a highly fluorescent gold nanocluster (AuNC@BSA) with red emissions at 640 nm through the biomineralization of bovine serum albumin (BSA) (Xie et al., 2009). Moreover, the BSA coating retained the capability of AuNC@BSA surface modification and had the potential as a drug carrier. On this basis, Liu et al. introduced HA into AuNC@BSA by cationic cross-linking (AuNC@CBSA@HA), followed by loading PTX, indocyanine green (ICG), and NO donors to construct a diagnostic nanoplatforms for combined chemotherapy/photothermal treatment AuNC@CBSA-PTX-ICG@HA-NO, and studied the suppressive effect of the nanoplatforms on breast cancer tumor growth and metastasis. The addition of HA shielded the positive charge effect of AuNC@CBSA, which effectively prolonged the non-specific toxicity and circulation time of nanoparticles *in vivo* and endowed the active targeting of CD44 receptor of nanoparticles as well as increased the penetration of nanoparticles while degrading at tumor sites. Besides, the nanoparticles showed significantly enhanced penetration ability for breast cancer cells by the synergistic effect of hyaluronidase degradation and NO regulation of the tumor microenvironment. Under the effect of combined treatment by delivering PTX and ICG, AuNC@CBSA-PTX-ICG@HA-NO3 exhibited excellent inhibition of *in situ* breast cancer tumor growth (∼95.3%) and lung metastasis (∼88.4%), providing an efficient potential strategy for the treatment of breast cancer (Liu et al., 2018b).

#### 4.1.3 Superparamagnetic iron oxide nanoparticle

Superparamagnetic iron oxide nanoparticles (SPIONs) are generally used in cancer treatment and diagnosis, with biodegradability and excellent magnetic properties (Kandasamy and Maity, 2015). SPIONs can achieve targeting capabilities in drug delivery utilizing magnetism and antibody attachment (Laurent and Mahmoudi, 2011). By combining HA and SPION(IO), Cheng et al. prepared a CuQDA/IO@HA nanoparticle loaded with dextran aldehyde (DA)-modified quercetin (Q)-Cu(II) complex with dual targeting function (HA-CD44 and magnetic navigation). CuQDA/IO@HA showed significantly higher internalization efficiency in BRCA mutant TNBC HCC1395 cells than in the non-targeted experimental group in the presence of CD44 receptor and magnetic navigation. In addition, CuQDA/IO@HA exhibited low toxicity to triple-negative breast cancer cells MDA-MB-231 (without BRCA mutation) while exhibiting high toxicity to HCC1395 cells, demonstrating the high specificity of the nanoparticles. In the mutant BRCA1 mice model, the dual targeting function increased the accumulation of CuQDA/IO@HA at tumor sites, reduced systemic toxicity, induced substantial DNA damage *via* Cu(II), and inhibited poly (ADP-ribose) polymerase (PARP), increased double-strand breaks (DSBs) accumulation and inhibited tumor growth. CuQDA/IO@HA provides a novel chemotherapy-free, drug-free regimen for breast cancer treatment through Cu(II)-induced DNA damage in breast cancer cells and further inhibition of DNA repair by quercetin (Cheng et al., 2021).

#### 4.1.4 Graphene oxide

Graphene oxide (GO), an oxidized derivative of graphene, has been widely used as a carrier to deliver drugs due to its high specific surface area and easy functionalization (Depan et al., 2011). Recently, GO has been shown to have an inhibitory effect on the metastasis of TNBC cells (Basu et al., 2018). Basu et al. modified metformin-loaded GO by PEG-PLGA and attached polymer nanoparticles to HA to obtain HA-GO-Met nanoparticles with cellular targeting of TNBC. In particular, HA provided CD44 receptor targeting to improve the uptake of breast cancer cells, while GO offered a higher efficiency encapsulation platform for the drug. The experimental data demonstrated that HA-GO-Met nanoparticles induced apoptosis, suppressed migration and reduced stem cells in MDAMB-231 cells by limiting the translocation of nuclear transcription factor NFkB-p65 in the nucleus and targeting miR-10b in a 4T1 murine mammary carcinoma model, with excellent anti-cancer effects without significant toxicity to normal cells. To summarize, the HA-GO-Met nanoparticles provide a new avenue for treating TNBC as a promising drug delivery platform to suppress the development of breast cancer cells (Basu et al., 2021).

#### 4.1.5 Carbon dots

Carbon dots (CDs) are novel nanomaterials with favorable biocompatibility and potential for surface modification (Singh et al., 2017). In recent years, CDs have received growing interest as drug delivery vehicles in cancer therapy (Panwar et al., 2019). Unfortunately, CDs cannot bind specifically to tumors; therefore, they cannot independently perform the task of effective drug delivery to tumor cells. Li et al. provided a facile strategy to construct a CD44-targeted carbon-site drug delivery system by a one-step method utilizing HA and loading DOX *via* a pH-responsive linker (HA-CD@p-CBA-DOX). The presence of HA conferred better structural stability and hemocompatibility to HA-CD@p-CBA-DOX and showed high cytotoxicity against 4T1 cells through CD44 receptor targeting and acid-sensitive DOX release. HA-CD@p-CBA-DOX exhibited excellent antitumor effects compared to free DOX without significant side effects. These results suggest that HA-CD@p-CBA-DOX deserves further study as a promising novel nano-drug delivery system for breast cancer treatment (Li et al., 2020).

### 4.2 HA hybrid with organic polymers

#### 4.2.1 Natural polymers

Chitosan (CS) is one of the most commonly used natural biopolymers with good biocompatibility and degradability (Elieh-Ali-Komi and Hamblin, 2016). CS and its derivatives are self-positively charged, so they are usually assembled with negatively charged polymers to form nanocomplexes, and HA is suitable for the need for a negative charge (Srivastava and Purwar, 2017). Based on these advantages, Wang et al. developed multifunctional polysaccharide-based nanocomplexes by employing a layer-by-layer (LbL) self-assembly method using an anionic aldehyde-functionalized hyaluronic acid (AHA), cationic hydroxyethyl chitosan (HECS) and targeting ligand human epithelial growth factor receptor 2 (HER2) antibody-decorated AHA (HA^HER2^) to prepare multifunctional polysaccharide-based nanocomplexes, and to chemically couple DOX and the photosensitizing prodrug 5-aminolevulinic acid (ALA) to AHA *via* Schiff base bonding to synthesize DOX/ALA- AHCS/HA^HER2^ nanocomplexes. The nanocomplex significantly enhanced the uptake of breast cancer MCF-7 cells through the active targeting of CD44 and HER2 antibodies. Furthermore, ALA in the nanocomplex could be effectively converted into endogenous photosensitizer PpIX in MCF-7 cells and achieve pH-responsive rapid release of DOX in the acidic microenvironment of cancer cells. Combining chemotherapy and photodynamic therapy (PDT) could effectively kill breast cancer MCF-7 cells (Wang et al., 2019a). Although this chemotherapy/PDT combination showed sound anti-cancer effects, the nanocomplexes showed unsatisfactory stability under PDT and limited tissue penetration by excitation light, limiting the application of nanopolymers. Using CDDP instead of ALA, CDDP molecules were inserted into the nanopolymer by chelating with AHA carboxyl groups to obtain the new NP ^HER2^ (DOX/CDDP) nanopolymer. The introduction of CDDP by cross-linking improves the stability of nanoparticles, but the combination of CDDP and DOX can achieve excellent anti-tumor ability without the need for an external excitation light source, completely overcoming the previous drawbacks. The experimental results showed that NPHER2 (DOX/CDDP) uptake by MCF-7 cells was significantly enhanced by the active targeting of CD44 and HER2 receptors. Due to the micro-acidic environment inside cancer cells, DOX and CDDP acidic pH-sensitive release significantly increased the mortality rate of breast cancer MCF-7 cells. The synergistic treatment of DOX/CDDP exhibited higher toxicity to breast cancer MCF-7 cells compared to DOX release alone. In summary, *in vitro* trials demonstrated the potential of NP^HER2^ (DOX/CDDP) for the treatment of breast cancer tumors, and this novel nanoplatform is expected to be further used for synergistic combination chemotherapy in breast cancer (Wang et al., 2019b).

Besides the efficiency of breast cancer suppression can be improved by combining multiple drugs for chemotherapy, the introduction of MicroRNA34a (miR34a) to reverse the resistance of breast cancer (BCa) cells and treat with chemotherapeutic drugs has also been studied. Yang et al. introduced miR34a and DOX into conjugated linoleic acid (CLA)-modified CS NPs and modified HA on the surface to obtain a novel targeting nanosystem (CCMDH NPs). CCMDH NPs increase the targeting of breast cancer cells through CD44 receptor action and utilize the miR34a to reverse Dox resistance in BCa to enhance DOX anti-tumor effects. The experimental data showed that CCmDHNPs suppressed and facilitated apoptosis by modulating protein expression of B-cell lymphoma-2 (Bcl-2) and poly (ADP-ribose) polymerase (PARP), producing sound inhibitory effects on xenograft tumors in nude mice, as well as effectively suppressed invasion, metastasis, and adhesion of breast cancer cells by regulating the levels of E-cadherin, N-cadherin, MMP2, CD44, and Snail molecules. CCmDHNPs have shown good inhibitory effects on the growth and migration of anti-DOX metastatic BCa tumors, providing an effective and novel therapeutic strategy for breast cancer treatment (Yang et al., 2021).

Hormone therapy also plays a vital role in treating breast cancer, and testosterone is an essential drug for hormone-sensitive metastatic breast cancer (Boni et al., 2014). Rezaei et al. designed a hyaluronic acid-chitosan-lipoic acid nanoparticle (HACSLA-NPs) with CD44 receptor targeting and reduction-responsive drug release, loaded with 17α-Methyltestosterone (MT). The effect of nanoparticles on breast cancer cells was evaluated. In the presence of glutathione, MT could achieve rapid release. Additionally, MT/HACSLA-NPs exhibited better targeting and good inhibition of BT-20 breast cancer cells compared to MT/CSLA-NPs. MT/HACSLA-NPs provide a novel and simple strategy for testosterone inhibition of breast cancer progression (Rezaei et al., 2020). Zeatin is a natural amphiphilic protein contained in corn, with the advantages of good biocompatibility, biodegradability, low cost, and easy availability. Zeatin nanoparticles are widely used to encapsulate and deliver various bioactive molecules. (Chen et al., 2019). Meanwhile, the complexes/or couplings composed of maize protein and other natural materials are simple to prepare and have good physical and chemical stability, which provide the possibility to prepare advanced zein nano delivery systems (Luo and Wang, 2014). Studies have shown that honokiol (HNK) can inhibit the growth and metastasis of various tumors, but its poor hydrophilicity and low bioavailability have significantly limited its use in cancer treatment. (Zhang et al., 2015b; Banik et al., 2019). Zhang et al. combined zein and HA and loaded HNK to prepare hydrophilic HA-coated HNK-loaded zein nanoparticles (HA-Zein-HNK). With CD44 receptor-mediated endocytosis, HA-Zein-HNK had efficient 4T1 cell endocytic uptake, antiproliferative and pro-apoptotic abilities *in vitro*. In addition, HA-Zein-HNK upregulated E-calmodulin expression in breast cancer cells and simultaneously downregulated Vimentin expression, significantly attenuating the invasion and migration of 4T1 cells. In the 4T1 tumor-bearing mice model, HA-Zein-HNK demonstrated a superior ability to suppress tumor growth and metastasis without significant systemic toxicity during treatment. HA-Zein-HNK provides a novel and promising strategy for preparing simple and efficient nanoparticles from natural material complexes to treat breast cancer (Zhang et al., 2020b).

#### 4.2.2 Synthetic polymers

Poly (amino acid) and its derivatives have good biocompatibility, biodegradability, and ease of modification. Through hydrophobic, electrostatic phase, and hydrogen bonding interactions, these nano micelles generally exhibit high hydrophobic drug loading capacity or high hydrophilic ability to modify hydrophobic segments. More importantly, these micelles have pH-sensitive drug release capability to ensure precise drug release in tumor tissues (Xu et al., 2015). Liu et al. used L-histidine (PHIS) to encapsulate the antitumor immunomodulator R848 to prepare PHIS/R848 nanocores (PHIS/R848) and synthesized HA-DOX prodrugs through hydrazone bonds to coat them on the surface of PHIS/R848, to obtain HA-DOX/PHIS/R848 nanoparticles with dual pH sensitivity. In the tumor microenvironment (pH∼6.5), PHIS/R848 released R848 to induce macrophage activation and cytokine secretion to achieve immunomodulation; moreover, at pH 5.5 (pH of endo/lysosomes), the breakage of hydrazone bond allowed the rapid release of DOX to achieve toxic effects on cells. The results indicated that PHIS/R848 had the same superior immunomodulatory ability as free R848, and HA-DOX could significantly inhibit the growth of breast cancer cells (MCF-7 and 4T1) mediated by CD44 receptors. The pH-ordered response of HA-DOX/PHIS/R848 resulted in the efficient release of R848 and DOX to target breast cancer cells. In 4T1 tumor-bearing mice, HA-DOX/PHIS/R848 exhibited excellent tumor targeting ability and significantly enhanced tumor suppressive activity in synergy with immunomodulation and chemotherapy, effectively inhibiting breast tumor growth. HA-DO X/PHIS/R848 combined with immunotherapy and chemotherapy showed great potential in breast cancer treatment (Liu et al., 2018c).

**FIGURE 5:**
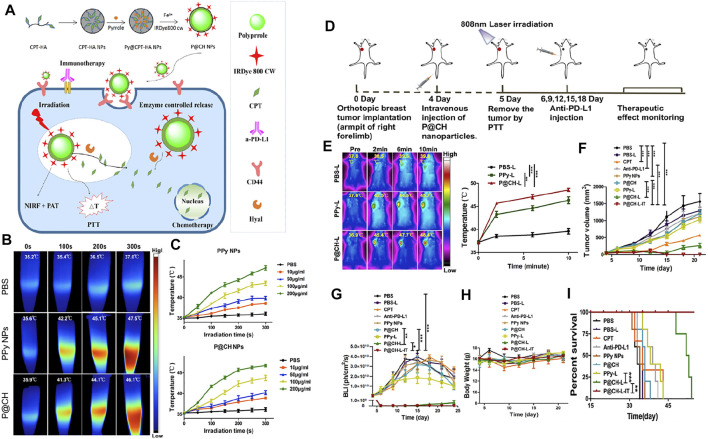
**(A)** Schematic illustration of the formation and functions of P@CH nanoparticles. **(B)** NIR thermal imaging before and after NIR irradiation on PBS, PPy NPs, and P@CH NPs plus laser irradiation. **(C)** Monitoring the increase of temperature with prolonged irradiation time over a series of concentrations of PPy NPs, and P@CH NPs (*n* = 3). **(D)** Scheme showing the experimental design to evaluate the therapeutic effect. **(E)** Infrared thermal images of the tumor-bearing mice at 24 h post-injection of PBS, PPy NPs, and P@CH NPs *via* the tail vein, before and after NIR laser light irradiation (1.5 W/cm2, 10 min), and the increase of temperature at tumor sites at different time points. **(F)** Tumor volume measurement for different treatment groups. **(G)** Quantification of the BLI signal of tumors after different treatments. **(H)** The body weight of 4T1 tumor-bearing mice with different treatments was measured. **(I)** Survival analysis of orthotopic 4T1 breast cancer mice with different treatments (*n* = 5) (**p < 0.01). Immune-memory effects induced by P@CH-L-IT combination treatment. Reproduced with permission from ref (Chen et al., 2019). CC BY 4.0. Copyright 2019 The Authors.

**FIGURE 6:**
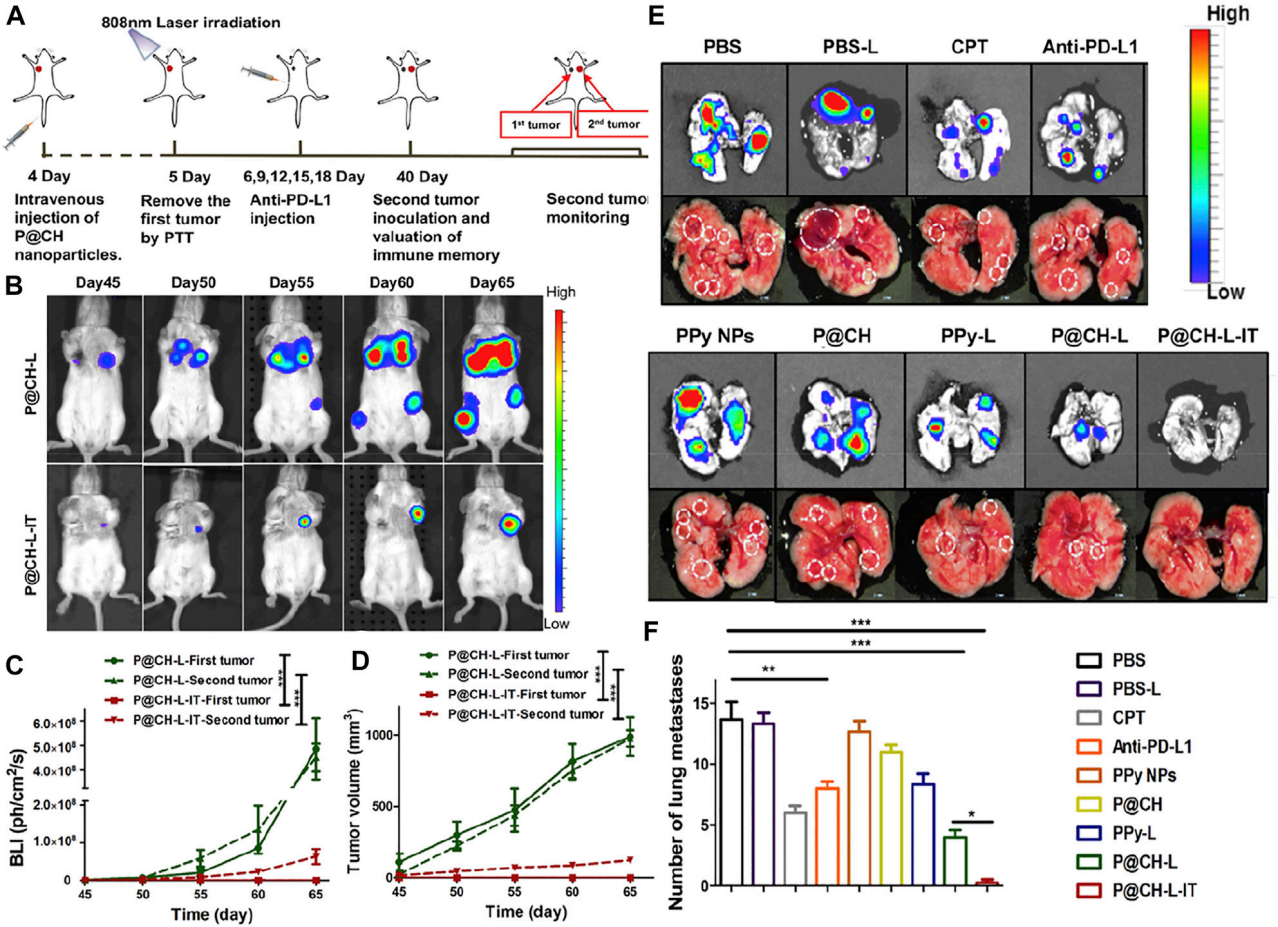
**(A)** Scheme showing the rechallenge experimental design to evaluate the immune-memory effect. **(B)** The dynamic BLI images of rechallenged mice from the P@CH-L and P@CH-L-IT groups (*n* = 5) for 65 continuous days. **(C)** Quantification of the BLI signal intensity of rechallenged tumors. **(D)** Tumor volume measurement of second rechallenged tumors and first tumors in two groups. Chemo-photothermal therapy in combination with immune checkpoint blockade therapy prevented lung metastases. **(E)** Representative BLI images and white-light pictures showing the lung metastases. **(F)** The number of lung metastasis nodules of different groups (*n* = 3). Reproduced with permission from ref (Chen et al., 2019). CC BY 4.0. Copyright 2019 The Authors.

Polypyrrole (PPy) has been innovatively applied to photothermal therapy for cancer due to its good biocompatibility and efficient photothermal conversion ability (Yang et al., 2012). Camptothecin (CPT) is a potent anticancer natural compound extracted from the bark of Camptotheca acuminata, which exerts antitumor activity by inhibiting DNA topoisomerase I (Topo I) (Tai et al., 2019). Sun et al. prepared a novel targeted PPy@CPT-HA-IRDye800CW (P@CH) nanoparticle with synergistic chemotherapy/photothermal therapy and PAI/FMI dual-modality imaging capability based on PPy and HA. P@CH NPs have a favorable PTT effect and can reach 46.1°C after 300 s of 808 nm NIR light irradiation. In the *in vivo* combination of photothermal therapy and immunotherapy against tumors, the P@CH + laser irradiation + anti-PD-L1 (P@CH-L-IT) group had surprising results compared to other experimental groups, where 4T1-fluc tumor bearing mice with *in situ* breast cancer tumors were completely eliminated and did not recur throughout the 24-day observation period, with mice surviving over 60 days, much longer than other treatment groups. In the tumor metastasis trial observation, all treatment groups had several pulmonary metastatic nodules, except for the P@CH-L-IT combination treatment group. In addition, the P@CH-L-IT group activated a systemic anti-tumor immune response to suppress lung metastasis and tumor recurrence in the long term. The combined therapy of P@CH-L-IT has an excellent effect on treating and preventing breast cancer recurrence. More excitingly, the PAI/FMI imaging properties of P@CH can be further used for image-guided chemoradiotherapy of breast tumors. This potential therapeutic strategy opens up a new path and direction for the clinical management of breast cancer and other malignant tumors. (Sun et al., 2019) (Figures 5, 6).

Polyethyleneimine (PEI), a cationic polymer, has been extensively studied as an anticancer drug delivery carrier (Fahira et al., 2022). α-naphthoflavone (ANF) is a CYP1B1 inhibitor that can reduce the multidrug resistance (MDR) of cancer cells to DTX by inhibiting the expression of CYP1B1 (Cui et al., 2015). Zhang et al. modified PEI with PLGA followed by wrapping HA outer layer by loading ANF and DTX to obtain HA/PEI NPs. Notably, HA/PEI NPs could effectively promote endocytosis of breast cancer cells by binding to CD44 receptors on the surface of breast cancer cells, thereby increasing the enrichment of ANF in breast cancer cells. The inhibitory effect of ANF on CYP1B1 expression increased the bioavailability of DTX and thereby enhanced the apoptosis-inducing ability of DTX. HA/PEI NPs effectively overcome the CYP1B1-mediated MDR in breast cancer and provide a promising strategy for the treatment of MDR breast cancer (Zhang et al., 2019).

D-ɑ-tocopheryl polyethylene glycol succinate (TPGS or Vitamin E TPGS) is approved by the FDA as a safe adjuvant. TPGS has the characteristics of excellent biocompatibility, improving drug solubility and permeability, and inhibiting the activity of ATP-dependent P-glycoprotein, so it is widely used in the delivery system of anticancer drugs (Guo et al., 2013; Yang et al., 2018b). Liu et al. prepared HA-based reduction-sensitive couples (HA-SS-DOCA) through previous work and synthesized mixed micelles together with TPGS, and used the mixed micelles to load DOX (D-MM) and quercetin (QU) (Q-MM) (Deng et al., 2017). Through the combined effect of Q-MM/Q-MM on MDA-MB-231/MDR1 cells, reducing the efflux of DOX by down-regulating the expression of P-glycoprotein can accelerate the mitochondrial apoptotic pathway and promote DOX-induced apoptosis. This combined treatment strategy improved tumor targeting in MDA-MB-231/MDR1 tumor-bearing mice and effectively inhibited tumor growth without serious side effects. In this study, the combination of DOX and QU effectively improved the bioavailability of DOX and reduced the toxic side effects, providing a novel strategy for chemotherapeutic drugs and chemotherapeutic drugs to reverse tumor MDR (Liu et al., 2020).

### 4.3 HA hybrid with organic molecule compounds

D-α-tocopherol succinate (TS) is a vitamin derivative with nontoxicity and good biocompatibility. Due to the poor water solubility of TS, it can be used as the hydrophobic core of self-assembled nanoparticles (Liang et al., 2015). In addition, TS induces apoptosis of malignant tumor cells through a reactive oxygen species (ROS)-dependent mechanism, which can synergize with chemotherapeutic drugs (Liang et al., 2015). Lee et al. designed to graft TS onto the HA backbone to prepare HA-TS amphiphilic nanoparticles, followed by introducing TPP as a mitochondrial targeting agent into HA-TS, and finally encapsulated lapatinib (LPT) to obtain HA-TS-TPP/LPT NPs for the treatment of breast cancer. HA-TS-TPP/LPT could effectively target tumors in MDA-MB-231 tumor-bearing mice through the EPR effect and CD44 receptor effect, and the cationic property and lipophilicity of TPP enhanced the intracellular accumulation of nanoparticles. In addition, after the nanoparticles were endocytosed by breast cancer cells, the mitochondrial targeting of TPP and the mitochondrial destruction capability of TS combined with LPT to produce killing effects on breast cancer cells, effectively improving the anti-cancer efficiency and anti-cancer activity of LPT. HA-TS-TPP/LPT showed promising effects in treating breast cancer in animal models, providing a possibility for further clinical research (Lee and Cho, 2018).

E-selectin (CD62E) is an inducible transmembrane adhesion protein expressed on endothelial cells activated by cytokines such as TNF-α and IL1-β during inflammation and tumors (Kang et al., 2016). In addition, E-selectin can target dividing microvascular endothelial cells that generate active blood vessels in tumor vasculature. Hence E-selectin can be used to modify nanoparticles to increase the uptake of dividing microvascular endothelial cells (Shamay et al., 2015). Han et al. applied E-selectin-binding peptide (Esbp) to treat breast cancer. The synthetic Esbp-polyethylene glycol-1-octadecylamine (Esbp-PEG-OA) divided microvascular endothelial cell-targeting ligand for amphiphilic HA-coupled prodrug micelles (HA-PTX). Esbp-PEG-OA and HA-PTX were self-assembled in an aqueous environment containing free DOX to obtain novel anticancer micelles (Esbp-HA-PTX/PTX). The experimental results showed that through the targeting of E-selectin and CD44 receptors, Esbp-HA-PTX exhibited both good targeting and excellent levels of cellular internalization on HUVEC and 4T1 cells *in vitro* and higher cytotoxicity compared to PTX solution. *In vivo*, Esbp-HA-PTX/PTX exhibited prolonged cycling time and excellent dual-cell targeting ability. More importantly, Esbp-HA-PTX/PTX exhibited excellent capability to inhibit tumor proliferation and metastasis without significant toxic side effects based on the suppression of intra-tumor microvasculature and tumor cell growth. Overall, Esbp-HA-PTX/PTX micelles provide a compelling new strategy for multicellular targeted breast cancer treatment (Han et al., 2017).

Among the treatments for breast cancer, endocrine therapy (anti-estrogen) is considered one of the preferable treatment modalities due to its low morbidity and mortality (Lumachi et al., 2011). Tamoxifen (TMX) is the preferred drug for anti-estrogen therapy for metastatic and advanced breast cancer, but long-term oral administration of TMX has significant side effects on the body (Hard et al., 1993). To overcome this challenging problem, we developed a self-emulsifying drug delivery system (SEDDS) for targeted delivery of TMX to the intestinal lymphatic system, reducing toxic side effects and increasing the bioavailability of TMX (Trevaskis et al., 2008). Batool et al. synthesized TMX-PAP-HA-SS-LCA SNEDDS using papain modification of S-protected hyaluronic acid-lithocholic acid co-block polymer carbodiimide containing TMX for suppression of breast tumors. Compared with the pure TMX experimental group, the mucus permeability of TMX-PAP-HA-SS-LCA was significantly improved, therefore enhancing cellular uptake. Additionally, TMX-PAP-HA-SS-LCA exhibited good compatibility with macrophages at different concentrations of TMX and had a lower drug release concentration on MCF-7 cells (IC_50_ = 5.98 ± 0.9 μg/ml, 24 h; 5.48 ± 1.4 μg/ml, 48 h) with targeting potential and antiproliferative solid activity. TMX-PAP-HA-SS-LCA improves the bioavailability of TMX through enhanced mucosal permeation without significant systemic toxicity, providing a novel and feasible strategy for endocrine therapy to inhibit the growth of breast tumors (Batool et al., 2020).

Among metal-organic frameworks (MOFs), zeolitic imidazolate framework-8 nanoparticle (ZIF-8) is frequently used as drug delivery carriers with good biocompatibility and ph-responsive degradability (Kida et al., 2013; Chen et al., 2020). Yu et al. loaded curcumin (Cur) into ZIF-8 (Cur@ZIF-8), prepared Cur@ZIF-8@HA nanoparticles by electrostatic interaction with HA, and applied it to breast cancer treatment. Benefiting from the excellent biocompatibility of HA and the active targeting ability of the CD44 receptor, Cur@ZIF8@HA showed a significant enhancement of biocompatibility and 4T1 cell uptake compared to Cur@ZIF-8. In addition, in 4T1 cells, Cur@ZIF8@HA could effectively induce apoptosis through LDH release, cell cycle arrest, and ROS overproduction. *In vivo*, tumor growth and lung metastasis could be effectively inhibited by 4T1 cell targeting of Cur@ZIF8@HA and the release of Cur in response to acidic pH. Cur@ZIF-8@HA showed excellent potential in breast cancer therapy and combining HA and MOF to prepare nanoparticles provides a new direction for developing anti-breast cancer drug delivery systems (Yu et al., 2021).

## 5 Clinical studies of nanomedicine in HA

HA has been extensively studied as a targeting ligand in the preclinical stage. HA-based NDDSs have shown enhanced anticancer activity and a higher safety profile than conventional regimens for cancer treatment. The classification of HA conjugates as new chemical entities (NCE) is one of the barriers to their treatment of cancer. Various anticancer drugs combined with HA for targeted therapy (e.g., irinotecan, DOX, 5FU, and MTX) have been studied in clinical trials. HA-irinotecan was shown to be biosafety and well-tolerated without reducing irinotecan drug activity in a phase 1 clinical trial of 12 patients (Brown, 2008). In another phase 2 clinical trial designed for 41 patients, the advantages of HA nanoformulations in progression-free survival and safety were demonstrated (Gibbs et al., 2011). In 2014, Alchemia Oncology initiated a study using HA-irinotecan in phase 2, a single-arm trial of FOLF(HA)iri in combination with cetuximab in second-line patients with KRAS wild-type metastatic colorectal cancer not treated with irinotecan (NCT02216487). The objective of the study was to confirm the safety and efficacy of FOLF(HA)iri plus cetuximab as a second-line treatment for patients with primary metastatic colorectal cancer treated with irinotecan.

## 6 Summary and prospective outlook

In recent decades, nanotechnology has been dramatically developed in cancer therapy. Presently, NDDSs play a critical role in the targeted delivery of breast cancer treatment, which can realize high accumulation and release of anticancer drugs at the tumor site to improve the therapeutic efficiency of drugs while reducing the toxic side effects on the normal tissues and organs of the human body, which not only overcomes the limitations of traditional drugs for breast cancer treatment but also has the potential to reduce the cost of conventional treatment, thereby solving a significant obstacle in breast cancer treatment.

Improving tumor-specific targeting is one of the critical factors for NDDSs to improve the efficacy of treating breast tumors. HA can target overexpressing breast cancer cells CD44 receptor, which is suitable as a potential target for breast cancer-targeting NDDSs. In addition, HA has good biocompatibility and excellent hydrophilicity, which provides advantages such as better hydrophilicity and long internal circulation time for inorganic or organic nanoparticles, including hydrophobic anticancer drugs. Therefore, HA is highly suitable for clinical application in breast cancer treatment.

In this review, we have discussed recent advances in treating breast cancer with HA-containing NDDSs. In recent years, many studies have shown that nano-delivery systems with HA can effectively target breast cancer cells *via* passive EPR and active CD44 receptor-mediated endocytosis, providing a simple and efficient way to target breast cancer cells for drug delivery actively. More excitingly, HA can be easily modified due to its wealthy chemical groups and can be combined with other inorganic or organic compounds to give HA NDDSs other advantages, such as increased drug loading, enhanced targeting, and some synergistic therapies. The HA NDDSs section of the review summarizes the progress of research on HA as a hydrophilic fraction for encapsulation or coupling of chemotherapeutic drugs, which directly enriches chemotherapeutic drugs at breast tumors through CD44 targeting, improving drug utilization and reducing systemic toxicity. In order to enhance the drug loading capacity and functionalization of HA NDDSs, researchers developed Modified HA NDDSs, which were further modified to confer higher drug loading capacity and tumor environment-sensitive drug release, significantly improving the anti-cancer effects of HA NDDSs. Many substances have anticancer potential, but their application is greatly limited because they do not possess the ability to target breast tumors or have poor biocompatibility on their own. HA nanohybrid NDDSs section summarizes the application of HA as an increase in the targeting ability or increased biocompatibility of other NDDSs, and the introduction of HA has dramatically broadened the choice of nano drugs in the treatment of breast cancer. They are resulting in Modified HA NDDSs and HA nanohybrid NDDSs with excellent performance for treating, preventing, and diagnosing breast cancer.

Unfortunately, until now, artificial mouse xenograft cancer models have been widely used due to the lack of suitable animal models, thereby having the disadvantage of not being able to mimic the different mutations carried by multiple cancer cells in human tumors, resulting in the inability to accurately mimic the immune landscape of *in situ* tumors (Sun et al., 2020). Although the development of HA-based drug carriers offers potential new clinical options for treating and preventing breast cancer, clinical studies demonstrating the efficacy of HA NDDSs in treating breast cancer are still lacking. In addition, in previous studies, there were discrepancies between preclinical and clinical experimental results of nano drugs, and nano drugs did not produce the same effects in humans as they did in rodents (Youn and Bae, 2018). However, the future work is still auspicious as more researchers from various disciplines (including physiology, pathology, oncology, material science, etc.) continue to develop superior NDDSs for better treatment of breast cancer in the clinic.

Nanomedicine is an emerging approach to cancer treatment, and a growing number of researchers continue to develop NDDSs with superior properties that offer more promising options for the treatment of breast cancer. Among these, HA-containing NDDSs are typically biocompatible and show significant potential for drug delivery and breast cancer tumor targeting. Although clinical data are lacking in breast cancer, HA has demonstrated its potential for clinical utilization in several clinical trials. These data provide strong evidence for continued research into the use of HA for future clinical breast cancer treatment.

## Glossary

AGP: α1-acid glycoprotein
AHA: Anionic aldehyde-functionalized hyaluronic acid
ALA: 5-aminolevulinic acid
ANF: α-naphthoflavone
BC: Breast cancer
BCSC: Breast cancer stem cells
Bcl-2: B-cell lymphoma-2
BSA: Bovine serum albumin
BF: Ibuprofen
CaP: Calcium phosphate
CDDP: Cisplatin
CDs: Carbon dots
CS: Chitosan
Ce6: Chlorin e6
CLA: Conjugated linoleic acid
CPT: Camptothecin
Cur: Curcumin
DA: Dextran aldehyde
DHA: Docosahexaenoic acid
DSBs: Double-strand breaks
DOX,DTX: Adriamycin, Doxorubicin
EPR: Enhanced permeability and retention effect
FA: Folic acid
FDA: Food and Drug Administration
GO: Graphene oxide
GNRs: Gold nanorods
GSH: Glutathione
HA: Hyaluronic acid
HJB: 17-hydroxy-jolkinolide B
HER2: Human epidermal growth factor receptor 2
HNK: Honokiol
IL-6: Interleukins
LPT: Lapatinib
MDR: Multidrug resistance
MOFs: Metal-organic frameworks
miR34a: MicroRNA34a
MSN: Mesoporous silica
MT: 17α-Methyltestosteron
NAP: Naproxen
NDDS: Nano drug delivery systems
PARP: Poly (ADP-ribose) polymerase
PEG: Polyethylene glycol
PEI: Polyethyleneimine
PDT: Photodynamic therapy
PHIS: L-histidin
PTT: Photothermal therapy
PPy: Polypyrrole
ROS: Reactive oxygen species
SEDDS: self-emulsifying drug delivery system
SFN: Sulforaphane
SHK: Shikonin
SPIONs: Superparamagnetic iron oxide nanoparticles
TNBC: Triple-negative breast cancer
TMX: Tamoxifen
TPGS or Vitamin E TPGS: D-ɑ-tocopheryl polyethylene glycol succinate
TPP: Riphenylphosphine
TS: D-α-tocopherol succinate
ZIF-8: Framework-8 nanoparticle
